# Tomato Derived Polysaccharides for Biotechnological Applications: Chemical and Biological Approaches

**DOI:** 10.3390/molecules13061384

**Published:** 2008-06-19

**Authors:** Giuseppina Tommonaro, Annarita Poli, Salvatore De Rosa, Barbara Nicolaus

**Affiliations:** Istituto di Chimica Biomolecolare, Consiglio Nazionale delle Ricerche (C.N.R.), Via Campi Flegrei, 34 80078 Pozzuoli (Napoli), Italy; E-mails: apoli@icb.cnr.it (A. Poli); sderosa@icb.cnr.it (S. De Rosa); bnicolaus@icb.cnr.it (B. Nicolaus)

**Keywords:** Tomato, solid wastes, cell culture, biotechnological application

## Abstract

Recent studies concerning the isolation and purification of exopolysaccharides from suspension-cultured tomato (*Lycopersicon esculentum* L. var. San Marzano) cells and the description of a simple, rapid and low environmental impact method with for obtaining polysaccharides from solid tomato-processing industry wastes are reported. Their chemical composition, rheological properties and partial primary structure were determined on the basis of spectroscopic analyses (UV, IR, GC-MS, ^1^H-, ^13^C-NMR). Moreover, the anticytotoxic activities of exopolysaccharides obtained from cultured tomato cells were tested in a brine shrimp bioassay and the preparation of biodegradable film by chemical processing of polysaccharides from solid tomato industry waste was also reported.

## 1. Introduction

Polysaccharides from natural sources have generated remarkable interest as biotechnological products due to their commercial uses in a wide range of industrial applications. Some of them, for example, showing strong antigenic and pathogenic activities, and are employed successfully by the pharmaceutical industry for the formulation of vaccines, while others are utilised as industrial food additives taking advantage of their useful physico-chemical (emulsifying, viscoelasticity, polyelectrolyte, adherence, bio-compatible, stabilizer, etc.) properties [1,2,3,4,5].

Polysaccharides, for their unusual multiplicity and structural complexity, contain many biological messages and accordingly they may perform several functions. Moreover, these biopolymers have the ability to interact with other polymers, such as proteins, lipids, as well as other polysaccharides [6,7,8].

Vegetables are the most important sources of polysaccharides (cellular walls or stock products). More recently, it has been seen that many microorganisms (bacteria and cyanobacteria) are also able to produce polysaccharides. The microbial polysaccharides are located in the cell wall (LPS), attached to the cells forming capsules (CPS) or secreted into the extracellular environment in the form of slime (exopolysaccharide, EPS) [9,10,11], but few data are available in literature regarding the polysaccharides from the cell cultures of *Solanaceae* [12]. We have previously reported the isolation and chemical characterization of the water-soluble bioactive polysaccharides from suspension-cultured cells of tomato (*Lycopersicon esculentum* L. var. San Marzano), and their anticytotoxic activities tested in a brine shrimp bioassay [13].

Furthermore we have reported our results concerning a rapid method conceived to recover high grade polysaccharides in high yields from solid tomato (*Lycopersicon esculentum* variety “Hybrid Rome”) processing industry wastes. The polysaccharides obtained from this natural and renewable source were characterised and used to prepare useful biodegradable film [14] and to obtain cheaper bacterial biomasses [15].

Waste management is a very important issue for the food industry, which is an important sector of the world economy. Besides the manipulation of fresh products, the new biotechnologies allow the recycling of wastes in order to obtain bio-products with high added value [16]. Tons of tomatoes are processed annually in the world by the food processing industry and the desiccation of the solid tomato waste produced represents an important approach to produce fertilizers; the goal of recovering biopolymers from such solid wastes (harmful to the environment and economically disadvantageous for the industry) represents an excellent alternative for their exploitation according to the new philosophies concerning sustainable industrial development [17].

## 2. Bioproducts from vegetal biomasses

Advances in genetics, biotechnology, process chemistry and engineering are leading to a new manufacturing concept for converting renewable biomass to valuable fuels and products, generally referred to as the 'biorefinery' [18].

Vegetables and their residual biomass enter the green biorefinery to be converted, by means of mechanical and biotechnological methods, into useful materials such as food and feed products and additives. Mediterranean plants are usually successfully extracted to give essential oils, resins and terpenes, by different methods, depending on the physico-chemical properties of the desired compounds, i.e. solubility, polarity, hydrophobicity, thermal stability and so on. These methods typically comprise water or organic solvent extraction (percolation, infusion, steam distillation, Soxhlet extraction), supercritical CO_2_ extraction, pressurized liquid extraction and microwave assisted extraction.

Potatoes and tomatoes, members of the *Solanaceae* plant family, serve as major, inexpensive low-fat food sources providing energy, high-quality protein, fiber, vitamins, pigments, as well as other nutrients. These crops also produce biologically active secondary metabolites, which may have both adverse and beneficial effects in the diet [19]. Nevertheless, other interesting bioproducts can be recovered from vegetable wastes, which are high value products employed in the agricultural, chemical and pharmaceutical fields. Current biotechnologies promote the use of solid wastes from vegetables to recover noble raw materials, thus avoiding sending them to the dump or to thermal destruction and thus allowing the recovery of high value added products, ecologically in line with the new philosophies concerning sustainable industrial development. Among the numerous biotechnological products recovered from vegetable wastes, the polysaccharides hold remarkable interest for their chemical-physical properties and for their wide range of biotechnological applications. Some of them, for example, are successfully employed by the pharmaceutical industry for the formulation of vaccines and for their anti-inflammatory and antioxidant activities. Moreover, the preparation of biodegradable and thermoplastic materials from these biopolymers using chemical processes is possible [14, 20]. Other interesting bioproducts potentially recoverable from vegetable wastes are antioxidant compounds (carotenoids, vitamins, polyphenols, etc), known for their ability to delay or to prevent the production of free radicals or to protect from their harmful effects [21]. In addition, vegetable wastes are useful as a substrate for bacterial growth, so cellular proteins could be recovered, indicating an alternative cheaper way to obtain microbial biomasses.

## 2. Exopolysaccharides from tomato cell culture

The cell culture of *Lycopersicon esculentum* L. var. San Marzano produced two main water soluble exopolysaccharides and the presence of extracellular polysaccharides was observed from the high viscosity of the culture media. The callus was induced from a sterile plant explant of *L. esculentum* cultured on MS basal medium, as reported by De Rosa *et al*. [22]. Suspension cultures were obtained from the 4^th^ generation callus by transferring callus (*ca.* 3 g) into liquid medium 100 mL). Cultures were maintained at 24°C, 150 rpm, under continuous light. The polysaccharide fraction was collected from the culture medium of tomato suspension cells (1 L) after four weeks of growth. The cell suspension was filtered and the exopolysaccharide fraction (260 mg) was obtained by EtOH precipitation of cell free culture broth. The raw material, tested for sugar content (70%), protein content (10%) and nucleic acid content (1 %), was purified by gel chromatography (Sepharose CL-6B DEAE) with a yield of 89%; the resulting compounds comprised three different fractions **EPS(1)** 9%, **EPS(2)** 60% and **EPS(3)** 31%, all containing less than trace amounts of protein and nucleic acids. **EPS(1)**, representing the neutral fraction, was eluted in H_2_O, while **EPS(2)** and **EPS(3)**, representing the acidic fractions, were eluted at different salt concentrations (0.3 and 0.4 M NaCl, respectively).

The mixtures of sugars (native and carboxyl reduced) in each fraction were identified by High Pressure Anion Exchange-Pulsed Amperometric Detection (HPAE-PAD) of the hydrolysed polysaccharides and by GLC and GC-MS of the corresponding alditol acetates and methyl glycoside acetates. The sugar analysis of the native EPSs indicated that **EPS(1)** was composed of arabinose/galactose/glucose/mannose in a relative ratio of 0.7:1.0:0.4:0.9; **EPS(2)** was composed of arabinose/galactose in a relative ratio of 0.3:1.0, and **EPS(3)** was composed of arabinose/mannose in a relative ratio of 1.0:0.5, respectively. The sugar analyses results of the methyl glycoside acetates indicated that **EPS(2)** was constituted of L-arabinose/D-galactose/L-arabinuronic acid (0.5:1.0:0.2, respectively) and **EPS(3)** of L-arabinose/D-mannose/L-arabinuronic acid (0.5:0.3:1.0, respectively).

**Figure 1:**
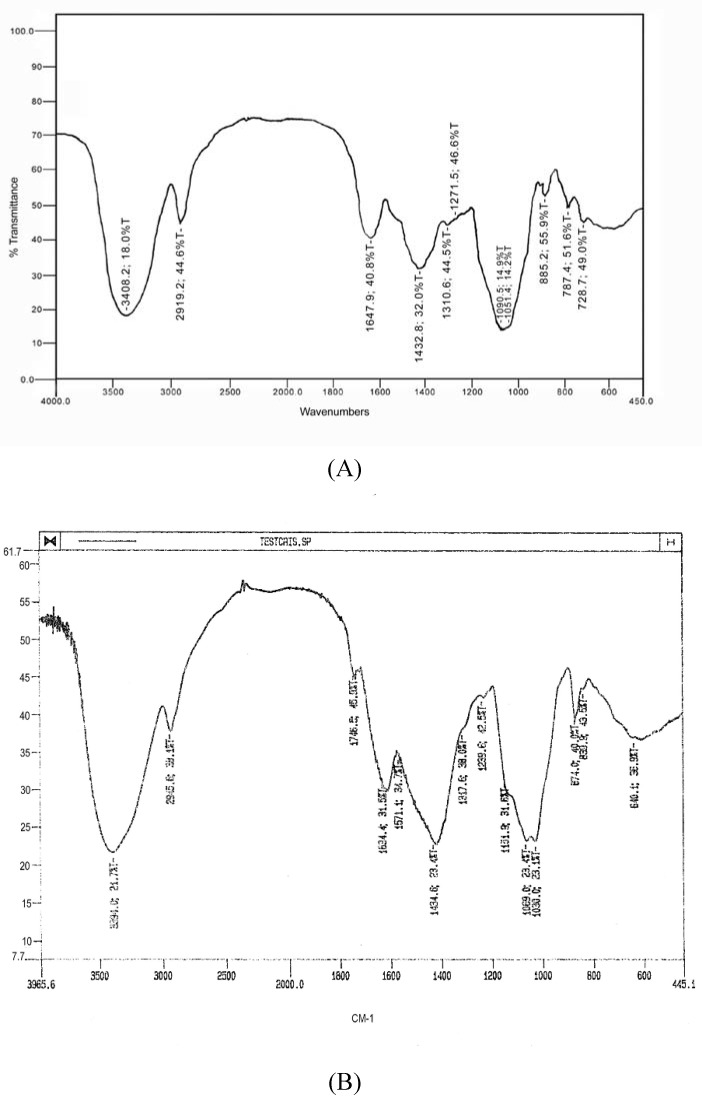
Infrared spectra of **EPS(2)** (A) and **EPS(3) **(B).

* The Fourier Transform Infrared spectrum was recorded using a Perkin-Elmer Paragon 500 single-beam spectrophotometer. The powdered sample was ground with KBr, the resulting disk placed under the beam and the spectrum was collected after 16 scans under nitrogen.

Only **EPS(2)** and **EPS(3)** were further analysed because **EPS(1)** was less pure and was recovered in a low yield. **EPS(2)** and **EPS(3)** were analysed by chemical and spectroscopic analysis. The quantities of uronic acid [23] varied with different preparations, reaching 170 μg/mg [**EPS(2)**] and 240 μg/mg [**EPS(3)**], respectively The specific rotations ([α]^25^_D_, concentration of 5 mg/mL H_2_O) of **EPS(2)** and **EPS(3)** were –50.40 and –60.80, respectively. The absolute configurations of the carbohydrates, determined by GLC of their acetylated (+)-2-butyl glycosides using optically active (+)-2-butanol, as described by Leontein *et al*. [24], was shown to be D-Gal and L-Ara for **EPS(2)** and D-Man and L-Ara for **EPS(3)**. 

The molecular weights of the EPSs were estimated from a dextrans standard calibration curve obtained by gel filtration on Sepharose CL-6B and also by density gradient centrifugation. In both methods, the molecular weights were approximately 8.0 x 10^5^ Da for **EPS(2)** and 9.0 x 10^5^ Da for **EPS(3)**.

The infrared (IR) spectra of EPSs were similar to those of bacterial polysaccharides [25]. A broad absorption band attributable to OH was observable at 3400 cm^-1^. The absence of sulfate groups in **EPS(2)** as well as in **EPS(3)** was confirmed by IR spectra and also by a negative colour reaction with sodium rhodizonate (Figure 1).

Analysis of the partially methylated alditol acetates, obtained from the permethylated EPSs after acid hydrolysis, showed the presence in both polysaccharides of hexose chains linked on C1-C2, C1-C3 and C1-C6 and side chains on C1-C2-C6 (Table 1)

**Table 1:** GC-MS of EPS(2) and EPS(3) from tomato cells of *Lycopersicon esculentum* var. San Marzano.

EPS(2)			
t_r_(min)	Sugar	% area	Link position
9.8	2,3,4,6-tetra-OMe hexose	17.43	Terminal hexose bonds at C1
11.98	3,4,6-tri-OMe hexose	27.96	Internal hexose bonds at C1 and C2
12.44	2,4,6-tri-OMe hexose	7.08	Internal hexose bonds at C1 and C3
12.90	2,3,4-tri-OMe hexose	17.37	Internal hexose bonds at C1 and C6
15.4	3,4-di-OMe hexose	27.96	Branching hexose bonds at C1,C2 and C6
**EPS(3)**			
**t_r_(min)**	**Sugar**	**% area**	**Link position**
9.8	2,3,4,6-tetra-OMe hexose	39.23	Terminal hexose bonds at C1
12.0	3,4,6-tri-OMe hexose	18.91	Internal hexose bonds at C1 and C2
12.4	2,4,6-tri-OMe hexose	7.36	Internal hexose bonds at C1 and C3
12.90	2,3,4-tri-OMe hexose	5.70	Internal hexose bonds at C1 and C6
15.4	3,4-di-OMe hexose	28.8	Branching hexose bonds at C1,C2 and C6

Methylation of the polysaccharides was carried out according to Manca *et al*. [25]. The methylated material (0.5 mg) was hydrolysed with 2 M trifluoroacetic acid (TFA) at 120°C for 2 hr and then transformed in partially methylated alditol acetates by reduction with NaBH_4_, followed by acetylation with Ac_2_O-pyridine (1:1) at 120°C for 3 hr. Unambiguous identification of sugars was obtained by Gas-Chromatography Mass Spectroscopy (GC-MS) using sugar standards. GC-MS was performed on a Hewlett-Packard 5890-5970 instrument equipped with an HP-5-MS column and with an N_2 _flow of 50 mL min^-1^; the programme temperature was: 170°C (1 min), from 170° to 250°C at 3° C min^-1^; t_r_ = retention time.

**Figure 2:**
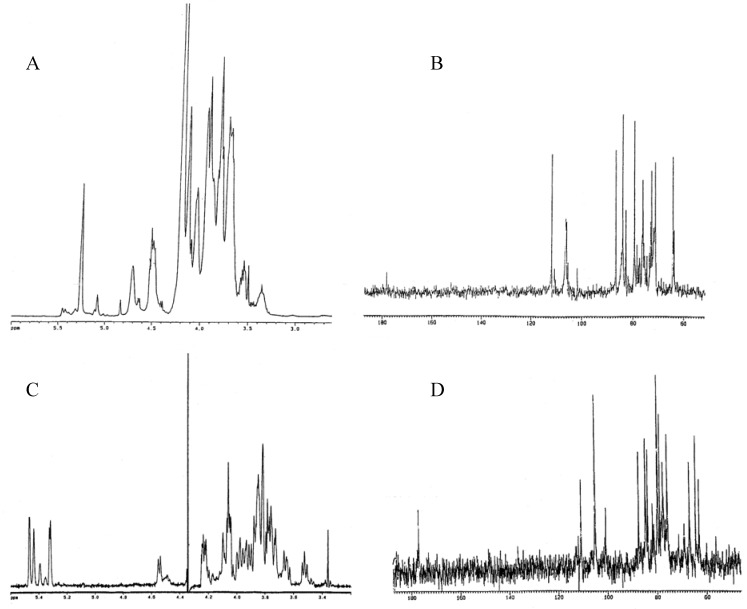
^1^H-NMR spectra of EPS(2) and EPS(3), panels A and C, respectively. ^13^C-NMR spectra of EPS(2) and EPS(3), panels B and D, respectively.

The ^1^H- and ^13^C-NMR spectra (*δ* chemical shifts are expressed in part per million, ppm, Figure 2) recorded in H_2_O at 70°C were quite complex. In the non anomeric proton region, several overlapping spin systems were evident. The ^1^H-NMR spectrum of **EPS(2)** showed, in the anomeric region, four major signals at *δ* 5.26 (1H, d, *J* = 3.0 Hz), 4.71 (1H, d, *J* = 1.5 Hz), 4.52 (1H, d, *J* = 8.0 Hz) and 4.49 (1H, d, *J* = 8.3 Hz) (Table 2). The ^1^H-NMR spectrum of **EPS(3)** showed five major anomeric signals at *δ* 5.47 (1H, d, *J* = 2.9 Hz), 5.44 (1H, d, *J* = 1.5 Hz), 5.39 (1H, d, *J* = 2.0 Hz), 5.32 (1H, d, *J* = 3.9 Hz) and 4.55 (1H, d, *J* = 7.8 Hz) (Table 2). The ^13^C-NMR spectrum of **EPS(2)** showed four signals at δ 111.9, 106.0, 106.3 and 106.4 in the anomeric region, confirming the presence of four residues in the repeating unit, and a small signal at δ 178.4 (COOH), due to the presence of uronic acid in minute quantities. The ^13^C-NMR spectrum of **EPS(3)** showed the presence of five signals at δ 111.5, 110.7, 105.1, 105.0 and 100.9, confirming the presence of five residues in the repeating unit, and an intense signal at δ 176.9 (COOH), indicative of the presence of uronic acid.

NMR spectra were recorded at 70°C on a Bruker AMX spectrometer (at 500 and 125 MHz for ^1^H- and ^13^C-, respectively). Samples were exchanged twice with D_2_O with intermediate lyophilization and then dissolved in 500 μL of D_2_O to a final concentration of 40 mg/mL. Chemical shifts are reported in ppm relative to sodium 2,2,3,3-*d*_4_-(trimethylsilyl)propanoate for ^1^H- and CDCl_3_ for ^13^C-NMR spectra.

The ^1^H- and ^13^C-NMR chemical shifts and the C-H coupling constants of each anomeric carbon were assigned by HMQC experiments [26]. Sugar residues were labelled from A to D for **EPS(2)** and from A to E for **EPS(3)**, in decreasing order of proton chemical shifts. The comparison of these values (Table 2) gave information about the anomeric configuration of some residues. In the ^1^H-NMR spectrum of **EPS(2)** the signal of the residue B at δ 4.71 was a *β*-manno (*J* = 1.5 Hz) while the signals at δ 4.52 and 4.49 of the residues C and D were typical of a *β*-gluco/galacto (*J* = 8.0-8.3 Hz) configuration. In the ^1^H-NMR spectrum of **EPS(3)** an *α*-manno configuration (*J* = 1.5 Hz) for residue B at *δ* 5.44, an *α*-gluco/galacto one at *δ* 5.32 for residue D (*J* = 3.9 Hz) and a *β*-gluco/galacto (*J* = 7.8 Hz) configuration at *δ* 4.55 for residue E were observed. The downfield ^13^C chemical shift observed for residues A of both **EPS(2)** and **EPS(3)** and residue C of **EPS(3)** may be indicative of a furanosidic form instead of a pyranosidic one. This was also confirmed by the presence of signals belonging to ring carbons in the region at δ 88-80 ppm, attributable to an arabino furanosidic residue present in both exopolysaccharides.

From these data, both polysaccharides showed a very complex primary structure. **EPS(2)** resulted to be a heteropolysaccharide with a tetrasaccharide repeating sugar unit whose residue configurations are *α*-manno, *β*-manno and *β*-gluco/galacto (1, 1 and 2, respectively); **EPS(3) **was a heteropolysaccharide with a pentasaccharide repeating sugar unit having *α*-manno, *α*-gluco/galacto and *β*-gluco/galacto residue configurations (3, 1, 1, respectively).

**Table 2:** Chemical shifts^a^ and coupling constants^b^ of anomeric signals in the ^1^H- and ^13^C-NMR spectra of EPSs from tomato cells of *Lycopersicon esculentum* var. San Marzano.

	EPS(2)			EPS(3)		
Residue	^a^δ H-1/C-1	^b^J_H-1,H-2_	^b^J_H-1,C-2_	^a^δ H-1/C-1	^b^J_H-1,H-2_	^b^J_H-1,C-2_
A	5.26/111.9	3.0	175.05	5.47/110.7	2.9	172.5
B	4.71/106.3	1.5	161.4	5.44/100.9	1.5	163.4
C	4.52/106.0	8.0	n.d.	5.39/111.5	2.0	176.0
D	4.49/106.4	8.3	n.d.	5.32/105.1	3.9	168.3
E				4.55/105	7.8	170.6

NMR spectra were recorded at 70°C on a Bruker AMX spectrometer (at 500 and 125 MHz for ^1^H and ^13^C, respectively). Samples were exchanged twice with D_2_O with intermediate lyophilization and then dissolved in 500 μL of D_2_O to a final concentration of 40 mg/mL. ^a ^Chemical shifts are reported in parts per million (ppm) relative to sodium 2,2,3,3-*d*_4_-(trimethylsilyl) propanoate for ^1^H- and CDCl_3_ for ^13^C-NMR spectra. Sugar components of **EPS(2)** are labelled from A to D, and those of **EPS(3)** are labelled from A to E, in both cases with decreasing chemical shifts. ^b ^Coupling constants are in Hz. n.d. = not detected.

We have also studied the effect of the exopolysaccharides produced by tomato suspension cultures on the inhibition of the cytotoxic effects produced by avarol. The ability of the exopolysaccharides obtained in this study to induce inhibition of avarol (10 μg/mL) toxicity was tested in the brine shrimp (*Artemia salina*) bioassay. Avarol is a sesquiterpene hydroquinone which showed strong toxicity (LC_50 _0.18 μg/mL or 0.57 nM) in brine shrimp bioassay, which gives results that correlate well with cytotoxicity in cancer cell lines such as KB, P388, L5178y and L1210 [27]. **EPS(2)** was a potent anticytotoxic compound in this bioassay; in fact, the inhibition of avarol toxicity of 50% (IC_50_) was observed at a concentration of 3 and 11 μg/mL for **EPS(2)** and **EPS(3)**, respectively.

## 3. Polysaccharides from solid tomato processing industry wastes

The extraction of polysaccharides from solid tomato processing industry wastes, produced by mechanical tomato pressing for the production of pulp and puree, was performed as described by Strazzullo *et al*. [14]. Starting from 20 g of tomato raw material (peels and seeds, rotten and unripe tomatoes), 1.5 g of sample **A **was obtained and chemically characterized.

The HPAE-PAD analysis performed on sample **A**, after hydrolysis with 2N TFA, (120°C for 2 hr), showed the following neutral sugar composition: glucose/xylose/galactose/galactosamine/ glucosamine/fucose in a relative molar ratio of 1:0.9:0.5:0.4:0.2:*tr*; its carbohydrate content was 100 % and the uronic acid content was 20 %, with galacturonic acid being the major uronic acid detected in the sample. Moreover, the sample was protein free. The chromatographic elution profile of the polysaccharides on Sepharose CL-6B, using a calibration curve of standard dextrans, indicated a molecular weight of 1 x10^6^ Da; Sample **A** has [α]^25^_D_ values of – 0.18 at concentration of 1 mg mL^-1^ in H_2_O.

**Table 3:** ^1^H-NMR data^a^ for the anomeric region of the spectrum of tomato waste Sample **A.**

Type^b^	δ^1^H	Multiplicity	J_1-2_^c^	Configuration
A	5.30	pseudo s	0.5-1 Hz	manno
B	5.27	D	3.8-4.0 Hz	gluco-galacto
C	5.26	D	3.8-4.0 Hz	gluco-galacto
D	5.18	pseudo s	0.5-1 Hz	manno
E	5.09	D	3.8-4.0 Hz	gluco-galacto
F	5.07	pseudo s	0.5-1 Hz	manno
G	5.06	D	3.8-4.0 Hz	gluco-galacto
H	4.94	D	3.8-4.0 Hz	gluco-galacto

^a ^Bruker AVANCE 400 MHz; sample was exchanged twice with D_2_O with intermediate lyophilization and then dissolved in 500 μL of D_2_O to a final concentration of 30 mg/mL, *δ* values (ppm) referred to sodium 2,2,3,3-*d*_4_-(trimethylsilyl) propanoate. ^b ^Labels refer to different monosaccharides, regarding type of glycosidic linkage position. ^c ^Coupling constant.

The^ 1^H-NMR spectrum of sample **A** (Table 3) showed a complex profile. The anomeric region of the spectrum (from *δ* 4.5 to δ 5.5) exhibited eight peaks; five of them well resolved doublets (d) with the same coupling constant value of *J*_1-2 _(3.8-4.0 Hz), probably due to a gluco-galacto sugar configuration; three other anomeric peaks, almost singlets with a small *J*_1-2 _(0.5-1 Hz), indicated the occurrence of a *manno* configuration; the upfield region of the spectrum showed a doublet peak at *δ* 1.20 indicative of the presence of deoxy-sugars in the polysaccharide. The eight anomeric signals indicated the presence of eight different monosaccharides, with regards to type or glycosidic linkage position. These monosaccharides were labelled A to H with respect to increasing *δ*. On the base of the chemical shifts and coupling constant data residues A, D, F have an *α*-manno configuration; B, C, E, G, an *α*-gluco-galacto configuration and the H residue a *β*-gluco-galacto one.

**Figure 3:**
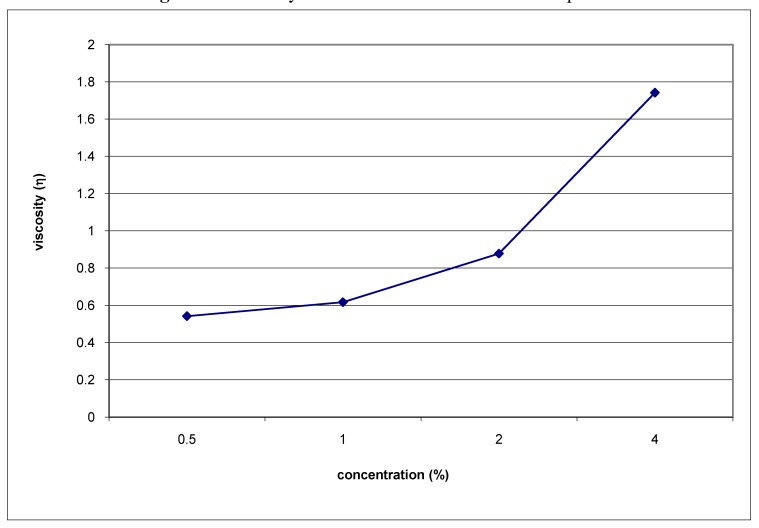
Viscosity/concentration correlation of Sample **A**.

The rheological properties were also characterized. The specific viscosity (η) of biopolymer **A** was measured for the aqueous solutions of polysaccharide at different concentration and pH values and resulted to be influenced by the size and number of macromolecules in solution. The specific viscosity was calculated by applying the following formula:

η = (t - t_0_ /t_0_)/C

where *t* is the time (*s*) employed from the polysaccharide solution to cover an established distance in the viscometer, *t*_0_ is the time (*s*) employed from the distilled water to cover an established distance in the viscometer, and *C* is the concentration (%) of the polysaccharide solution. As concentration increases coils start to overlap and become entangled, with viscosity showing a more marked dependence on concentration reaching η=1.7 at 4% of concentration (Figure 3). The viscosity does not change drastically respect to the increase of pH and its maximum value was obtained at pH 3.0 for a 1% polysaccharide solution in 50 mM citrate buffer (η=3.29).

The viscosity determination of aqueous solution of waste tomato polysaccharide at different concentration values were carried out using Cannon-Ubbelohde 75 suspended level viscometer at 25°C.

**Figure 4:**
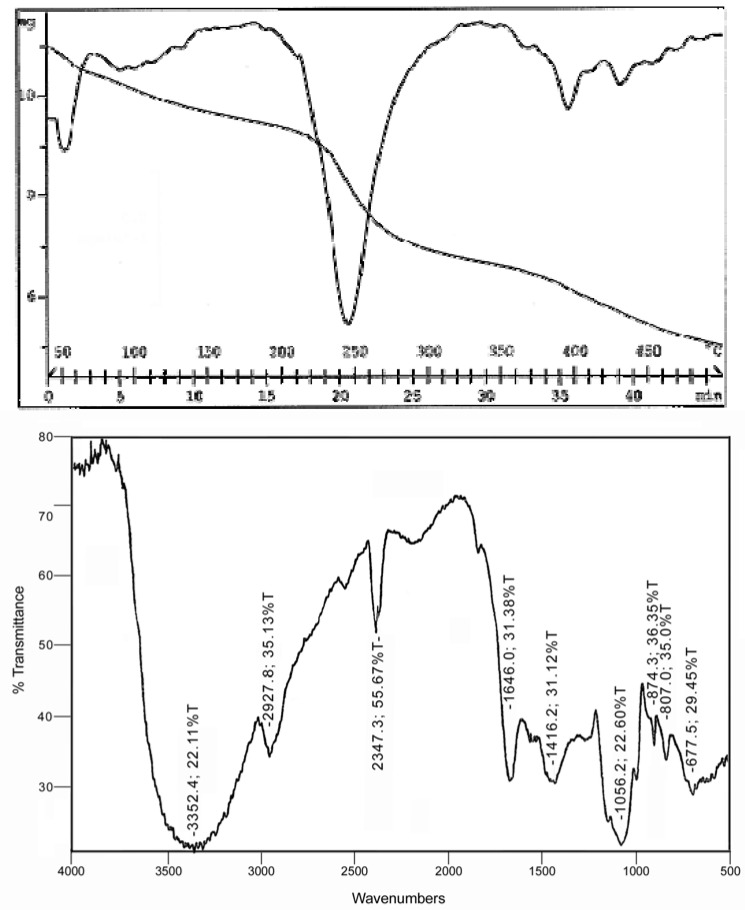
Thermogravimetric analysis (top) and Fourier Transform Infrared spectrum (bottom) of Sample **A**.

The degradation temperature of sample **A** (10 mg) was 250° C in 20 min, leaving a residue of about 5 mg (Figure 4A). Moreover, the infrared spectrum of this biopolymer showed the characteristic peak signals of polysaccharides: OH stretching at 3.400 cm^-1^, CH and C=O stretching at 2929 cm^-1 ^and 1730-1660 cm^-1 ^respectively. A SO group was absent, in fact no signals were detected at 1240 cm^-1 ^(Figure 4B).

*Top*: Thermogravimetric analysis of waste tomato sample **A** was performed using Mettler Toledo Star System equipped with a thermo analytical balance over a temperature range of 50° to 450°C with a temperature program of 10 min at 50°C followed by a 10°C/min ramp. *Bottom*: Fourier Transform Infrared spectrum of sample **A** recorded using a Perkin-Elmer Paragon 500 single-beam spectrophotometer. The powdered sample was ground with KBr, placed under the beam as a disk and the spectrum was collected after 16 scans under nitrogen.

Sample **A** was used for the preparation of biodegradable films by solubilizing 50 mg of polysaccharide in 5 mL of distilled water at room temperature and adding 5 mg of glycerol as plasticizer [28]. Films thus obtained were clear and elastic; solid and durable when recovered from small static deformations produced by the applied tensile stress.

**Figure 5:**
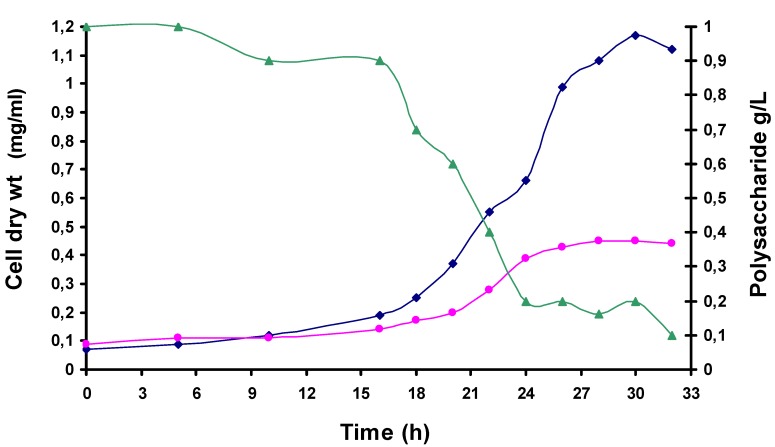
Biodegradability test of Sample **A** using thermohalophilic *Thermus thermophilus* strain.

Strain Samu-SA1 cell growth (expressed as dry cell weight for mL of culture broth, mg/mL) on polysaccharide medium (■-■-■) and on TH medium (●-●-●) at 75°C. The growth was followed by measuring absorbance at 540 nm and converted to the cell dry weight by an appropriate calibration. The depletion of polysaccharide reported as g/L followed using Dubois method (▲-▲-▲).

Finally, in order to verify the biodegradability of sample **A**, a growth test using a new thermohalophilic *Thermus thermophilus* strain (Samu-Sa1 strain) isolated from hot springs of the Mount Grillo (Baia, Naples, Italy) was performed [15]. This strain was grown on M162 medium [29] modified with 0.2% NaCl at pH 7.2 and using as sole carbon source 0.1% of tomato polysaccharide. After 30 h of batch incubation at 75°C (the optimal temperature of Samu-Sa1strain) the tomato polysaccharide was completely hydrolysed as resulted from phenol/sulphuric acid method tested on cell free cultural broth [30] (Figure 5). Moreover, the growth curves of strain on polysaccharide medium was 2.6 fold higher than that obtained in TH medium (standard medium) with a yield of 1.2 g of dry cells/L (Figure5).

## 4. Conclusions

Three exopolysaccharides **EPS(1)**, **EPS(2)**, **EPS(3)** were isolated from suspension-cultured *Lycopersicon esculentum* (var. San Marzano) cells. The partial primary structures, hypothesized on the basis of spectroscopic analyses, resulted in a peculiar complex primary structure for all exopolysaccharides. In particular **EPS(2)** was a heteropolysaccharide characterized by a tetrasaccharide repeating unit and **EPS(3)** was a heteropolysaccharide characterized by a pentasaccharide repeating unit. The anticytotoxic activities of exopolysaccharides, tested in a brine shrimp bioassay, showed a potential role in a host defense mechanisms and further studies are necessary to test other biological activities.

Food canning industries represent an important area of the Italian economy, in particular the industrial conversion of tomatoes into tomato purée, pulp and tomato minced from seed-firms. One of the main problems of food industry is the management of wastes and their conversion into higher added value products. Contemporary eco-compatible technologies promote the use of food waste to obtain biopolymers that can be re-used in the same sector as the raw materials. 

We have established that the method herein described is a rapid procedure affording high yield cell-wall polysaccharide production (7.5 %), with very low environmental impact. The sugar analysis of the polymers revealed the presence of glucose and xylose as major components, and a low level of uronic acids, in contrast to that of the cell-wall pectic polysaccharides that contain arabinose in large amounts and higher contents of uronic acids. These unusual findings are due to the different minor polysaccharides extracted by the use of these new methods. The data we have presented are appropriate for obtaining of minor polysaccharides with interesting chemical-physical properties such as high viscosity, high thermal resistance and high molecular weight from tomato waste material in high yield and therefore, they can be used in a better way than commercially available sources. The structure of the sample **A** was very complex and presented eight monosaccharides as repeating unit, three of them with a probable *α*-manno configuration, four residues with an *α*-gluco-galacto configuration and one residue showing a *β*-gluco-galacto configuration.

The main point of interest was the formation of biodegradable films on addition of glycerol using these bio-polymers. The film formed from sample **A** was durable and elastic and could be used in different fields such as agriculture, i.e., for protected cultivation in mulching operation techniques. In fact, the plastic material usually used for mulching and solarization have optimum mechanical characteristics and low cost but they are not biodegradable, not reusable, and their residues are discarded as a special waste.

An additional biotechnological use of the tomato polysaccharide could be as a cheaper substrate to obtain microbial biomasses. *Thermus thermophilus* strain Samu-SA1 possesses many hydrolytic enzymes with potential biotechnological applications and was able to grow on very cheap medium. In fact 1.2 g/L of dry cells were obtained when waste polysaccharide, extracted from discarded industrial tomato processing, was used as sole carbon source with a biomass yield of 2.6 fold higher than that obtained with standard medium [31].
